# Multi-modal deep learning improves grain yield prediction in wheat breeding by fusing genomics and phenomics

**DOI:** 10.1093/bioinformatics/btad336

**Published:** 2023-05-23

**Authors:** Matteo Togninalli, Xu Wang, Tim Kucera, Sandesh Shrestha, Philomin Juliana, Suchismita Mondal, Francisco Pinto, Velu Govindan, Leonardo Crespo-Herrera, Julio Huerta-Espino, Ravi P Singh, Karsten Borgwardt, Jesse Poland

**Affiliations:** Department of Biosystems Science and Engineering, ETH Zurich, Basel, Switzerland; Swiss Institute of Bioinformatics, Lausanne, Switzerland; Visium, Lausanne, Switzerland; Department of Plant Pathology, Kansas State University, Manhattan, KS, United States; Department of Agricultural and Biological Engineering, IFAS Gulf Coast Research and Education Center, University of Florida, Wimauma, FL, United States; Department of Biosystems Science and Engineering, ETH Zurich, Basel, Switzerland; Swiss Institute of Bioinformatics, Lausanne, Switzerland; Department of Machine Learning and Systems Biology, Max Planck Institute of Biochemistry, Martinsried, Germany; Department of Plant Pathology, Kansas State University, Manhattan, KS, United States; Global Wheat Program, International Maize and Wheat Improvement Center, Texcoco, Estado de Mexico, Mexico; Global Wheat Program, International Maize and Wheat Improvement Center, Texcoco, Estado de Mexico, Mexico; Global Wheat Program, International Maize and Wheat Improvement Center, Texcoco, Estado de Mexico, Mexico; Global Wheat Program, International Maize and Wheat Improvement Center, Texcoco, Estado de Mexico, Mexico; Global Wheat Program, International Maize and Wheat Improvement Center, Texcoco, Estado de Mexico, Mexico; Global Wheat Program, International Maize and Wheat Improvement Center, Texcoco, Estado de Mexico, Mexico; Campo Experimental Valle de Mexico-INIFAP, Texcoco, Estado de Mexico, Mexico; Global Wheat Program, International Maize and Wheat Improvement Center, Texcoco, Estado de Mexico, Mexico; Department of Biosystems Science and Engineering, ETH Zurich, Basel, Switzerland; Swiss Institute of Bioinformatics, Lausanne, Switzerland; Department of Machine Learning and Systems Biology, Max Planck Institute of Biochemistry, Martinsried, Germany; Department of Plant Pathology, Kansas State University, Manhattan, KS, United States; Center for Desert Agriculture, King Abdullah University of Science and Technology, Thuwal, Saudi Arabia

## Abstract

**Motivation:**

Developing new crop varieties with superior performance is highly important to ensure robust and sustainable global food security. The speed of variety development is limited by long field cycles and advanced generation selections in plant breeding programs. While methods to predict yield from genotype or phenotype data have been proposed, improved performance and integrated models are needed.

**Results:**

We propose a machine learning model that leverages both genotype and phenotype measurements by fusing genetic variants with multiple data sources collected by unmanned aerial systems. We use a deep multiple instance learning framework with an attention mechanism that sheds light on the importance given to each input during prediction, enhancing interpretability. Our model reaches 0.754 ± 0.024 Pearson correlation coefficient when predicting yield in similar environmental conditions; a 34.8% improvement over the genotype-only linear baseline (0.559 ± 0.050). We further predict yield on new lines in an unseen environment using only genotypes, obtaining a prediction accuracy of 0.386 ± 0.010, a 13.5% improvement over the linear baseline. Our multi-modal deep learning architecture efficiently accounts for plant health and environment, distilling the genetic contribution and providing excellent predictions. Yield prediction algorithms leveraging phenotypic observations during training therefore promise to improve breeding programs, ultimately speeding up delivery of improved varieties.

**Availability and implementation:**

Available at https://github.com/BorgwardtLab/PheGeMIL (code) and https://doi.org/doi:10.5061/dryad.kprr4xh5p (data).

## 1 Introduction

Food security is a critical issue for a growing global population (Food and Agriculture Organization of the United Nations 2019). To ensure appropriate food supply, it is of utmost importance to identify and grow the most robust, highest yield crop varieties. To that end, plant breeding programs are designed to identify the crossings that guarantee the highest yield while ensuring resistance and resilience across environments (Crossa *et al.* 2017). Several technological and methodological advances such as high-throughput phenotyping and genomic selection have enabled a constant growth in yield for various crops across the years (Tester and Langridge 2010): for example, breeders of wheat (*Triticum aestivum*) manage to obtain average annual gains of 1% (Tadesse *et al.* 2019). Nonetheless, demand for staple food raises steadily and more steeply, with an average annual demand increase of 1.7% for wheat (Tadesse *et al.* 2019). To sustain such a growing demand without needing to dedicate more land and while addressing less favorable environmental conditions caused by climate change, faster and more efficient breeding strategies need to be adopted.

Current breeding programs rely on end-point destructive measurements such as grain yield measurement to rank and select the best candidates. Thus full growth cycles are needed to reach a selection decision, hindering the speed of the program and limiting selection gains. Tackling these limitations can be achieved by predicting the yield of new breeding lines in a given environment before the end of the crop cycle, or even before field testing altogether by using genomic data. Yield prediction is a task that has been studied for decades (Parry *et al.* 2005). Proposed approaches can be grouped in (i) genomic predictions, for which the yield is predicted independently of the environmental conditions, and (ii) phenotype-based predictions, where the predicted yield is linked to a specific plant or plot and its observed aspects—which also encompass environmental conditions.

Several machine learning approaches were suggested for both approaches (van Klompenburg *et al.* 2020). For genomic predictions, existing techniques leverage single nucleotide polymorphism (SNP) data and rely on linear models (Ibba *et al.* 2020). However, historical models cannot account for environmental factors: most statistical genetics studies either try to control the environmental conditions (Bloom *et al.* 2013) or design experiments to minimize the impact of the environment (Poland *et al.* 2012), which results in suboptimal prediction performance. Recently, models have been proposed accounting for environmental data, both for linear (Millet *et al.* 2019) and for nonlinear models (Pérez-Enciso and Zingaretti 2019, Abdollahi-Arpanahi *et al.* 2020). Multitrait models (Arouisse *et al.* 2020) such as MegaLMM (Runcie *et al.* 2021) allow for the incorporation of phenotypic information to improve yield predictive ability. All evidence shows that accounting for environmental conditions unlocks higher predictive accuracy, but decoupling environmental factors from genomic signal for new predictions is not straightforward. Nonetheless, these methods do not scale with large sample sizes, which limit them in applications to large-scale breeding programs (Runcie *et al.* 2021).

For phenotype-based yield predictions, where predictive accuracies are much higher, the most common source of data is remote sensing data, being easy to collect and to access. Both satellite imagery and low altitude Unmanned Aerial Vehicle (UAV) embedded sensors are used, but the latter are preferred for their finer-grained resolution and high-throughput phenotyping abilities (Colomina and Molina 2014, Haghighattalab *et al.* 2016). Many crop yield prediction models based on UAV-acquired imagery were developed for a multitude of crops (Maresma *et al.* 2016, Du and Noguchi 2017, Gong *et al.* 2018). Traditional machine learning models were applied on manually crafted features (vegetation indices, VIs) derived from images (Heremans *et al.* 2015, Wu *et al.* 2015, Pantazi *et al.* 2016, Stas *et al.* 2016). More recently, deep learning techniques leveraged the potential of high-dimensional image data both across data sources (Herrero-Huerta *et al.* 2020, Maimaitijiang *et al.* 2020) and time (Khaki *et al.* 2019, Nevavuori *et al.* 2020). However, plot-based yield prediction relies on late-stage growth images for accurate predictions, saving only a limited amount of time in the breeding process. Moreover, none of these approaches allows for the integration of genetic information in the prediction task, limiting their predictive ability in new environments and for new sets of breeding lines.

Here, we propose PheGeMIL (**Ph**enotype-**Ge**notype **M**ultiple **I**nstance **L**earning), a flexible deep learning framework that uses multichannel, temporal inputs to predict grain yield with superior accuracy. PheGeMIL leverages attention mechanisms, a family of deep learning modules that are highly effective for modeling relationship in structured objects (Vaswani *et al.* 2017), to comprehensively and simultaneously take into consideration four sources of information: (i) longitudinal multispectral images, (ii) longitudinal thermal images, (iii) longitudinal digital elevation models, and (iv) genetic variants in the form of single nucleotide polymorphisms (SNPs). Our method effectively fuses genome-wide genetic variants and rich phenotypic observations reflecting the environment and its effects over time on the predicted phenotype. Furthermore, PheGeMIL, once trained on multiple channels, can be implemented for yield prediction from genotype alone, in any new environment, outperforming solid baselines and opening the way for improved applications of genomic selection in breeding programs. In the next section, we will describe our setting and method.

## 2 Materials and methods

### 2.1 Plant material and field layout

Spring wheat (*Triticum aestivum* L.) breeding lines of two different trials, named as Yield Trials (YT, 27°22′57.6″ N, 109°55′34.7″ W) and Elite Yield Trials flat planting (EYT, 27°23′0.1″ N, 109°55′7.9″ W), were evaluated in Cd. Obregon, Mexico in the International Maize and Wheat Improvement Center (CIMMYT) wheat breeding program. Both trials were sown in November 2017, during the 2017–2018 field season. The YT experiment consisted of 9596 spring wheat entries distributed in 320 trials, while the EYT experiment consisted of 1170 entries distributed in 39 trials. YT experiment was arranged following an alpha-lattice design and distributed within two blocks (replications). EYT has 39 trials connected through checks, each of which have an alpha-lattice design Krause *et al.* (2019). The YT plots served as experimental units and were 1.7 m × 3.4 m in size, planted on two raised beds spaced 0.8 m apart with paired rows on each bed at 0.15 m spacing for each plot. The EYT plots were sown in flat and were 1.3 m × 4 m in size with six rows per plot. Both experiments were flood irrigated to optimal soil moisture. Final crop yield was harvested with a small plot combined and measured on a per plot weight and converted to tons per hectare (t/ha) on a per plot basis.

### 2.2 Data acquisition and preprocessing

A DJI Matrice 100 (DJI, Shenzhen, China) UAS was used for data acquisition. It was equipped with a 5-channel multispectral RedEdge camera (MicaSense Inc., USA) with blue (475 nm), green (560 nm), red (668 nm), RedEdge (717 nm), and near infrared (840 nm) bands. Flights were conducted between 11 a.m. and 2 p.m. at a ground altitude of 35 m using the data collection procedures previously developed by the Poland Lab (Singh *et al.* 2019). To ensure highly accurate data, the acquired images were geo-referenced and geo-rectified using ground control points (GCPs) of bright white/reflective square markers uniformly distributed across the field area. To collect thermal images, a FLIR VUE Pro R thermal camera (FLIR Systems, USA) was carried by the DJI Matrice 100 and flights were performed at 60 m above the ground. Extraction of plot-level phenotypic values from orthomosaic and orthorectified images followed the methodology of Wang *et al.* (2020). All breeding lines were profiled using the genotyping-by-sequencing protocol of Poland *et al.* (2012) and sequenced on an Illumina HiSeq2000 or HiSeq2500. Single nucleotide polymorphism (SNP) markers were called from tag alignment to the Chinese Spring reference genome assembly v1.0 (International Wheat Genome Sequencing Consortium and Others 2014). Genotyping calls were extracted and filtered so that the percent missing data per marker was <40% and percent heterozygosity was <10%. Lines with more than 50% missing data were removed.

### 2.3 Model architecture

Multiple Instance Learning (MIL) aims at learning a target value from a sample that is a *bag* of instances. We propose a deep learning model to tackle the MIL task. More recently, for MIL, researchers adapted deep learning pipelines to the problem by using neural networks to learn useful low-dimensional representations of the single instances and aggregate them using permutation-invariant functions, such as attention mechanisms (Ilse *et al.* 2018). More formally, for one target variable y∈R, instead of a single associated instance $x\in R^{n}$ we have an associated *bag* of instances $X=\{x_{1},\ldots ,x_{k}\}$ that do not exhibit a particular ordering between each other. The learning problem then aims to learn $\hat{y}=S(X)$, where *S* is a function that is permutation-invariant to the elements in *X* (i.e. the output of *S* is not influenced by the ordering of $x_{1},\ldots ,x_{k}$). Zaheer *et al.* (2017) show that such a function needs to be decomposable in a sum of transformations as follows.

Theorem 1 *(*Zaheer *et al.* 2017). *A function S(X) for a set of instances X having countable elements is a valid set function (i.e. permutation-invariant to the elements of X), iff it can be decomposed in the formwhere g and f are suitable transformations.*

(1) $$S(X)=g(\sum_{x\in X}f(x)),$$

This allows us to view the MIL problem as a three-step process: (i) transform the instances with a function *f*, (ii) combine the transformed instances with a permutation invariant function ϕ (e.g. sum, average), (iii) transform the combined instances with a function *g*. In other terms, we obtain an *embedding* for each instance via *f*, combine them in an invariant manner with the pooling operator ϕ and rely on *g* to get a useful output for the learning task at hand. This process can be translated to a deep learning setting, where both *f* and *g* are neural networks and ϕ needs to be a differentiable pooling operator. Common pooling operators in deep learning include the maximum operator and the mean operator (Zaheer *et al.* 2017). Nevertheless, these pooling operators have the disadvantage of being predefined and nontrainable. That is why we prefer an attention-based pooling mechanism (Ilse *et al.* 2018), which offers a higher flexibility to the data and the tackled task as well as a certain degree of *interpretability* of the pooling.

Attention mechanisms have been extensively used in natural language processing (Vaswani *et al.* 2017), image captioning (Xu *et al.* 2015), and graph neural networks (Veličković *et al.* 2018). Broadly speaking, attention mechanisms are neural networks’ components in charge of quantifying the interdependence of input elements (i.e. weight the contributions of each input based on the input itself and on other inputs). This translates in finding weights $a_{i}=f(h_{1},\ldots ,h_{k})\;\;\forall i=0,\ldots ,k$, where $H=\left\{h_{1},\ldots ,h_{k}\right\}$ is the set of inputs. In their simplest form, they can be a simple inner product between the inputs $h_{i}$. More advanced attention mechanisms are composed by a neural network that learns the relative importance of each input element. Ilse *et al.* (2018) proposed a MIL pooling of the sort. Let $H=\left\{h_{1},\ldots ,h_{k}\right\}$ be the set of *k* embeddings of dimension *m* for a sample $X=\left\{x_{1},\ldots ,x_{k}\right\}$, then the pooling operation is given by: with where $w\in R^{l\times 1}$ and $V\in R^{l\times m}$. This pooling allows for more flexibility in the way the contribution of individual instances are combined, unlocking better prediction performance. Additionally, the weights $a_{1},\ldots ,a_{k}$ can be used to gauge the relative importance of each instance of the sample and provide interpretability around the model’s prediction.


(2) $$z=\sum_{i=1}^{k}a_{i}h_{i},$$

(3) $$a_{i}=\mathrm{softmax}{\left(w^{⊤}\text{tanh}(VH^{⊤})\right)}_{i}=\frac{e^{w^{⊤}\text{tanh}(Vh_{i}{}^{⊤})}}{\sum_{j=1}^{k}e^{w^{⊤}\text{tanh}(Vh_{j}{}^{⊤})}},$$

**Figure 1:**
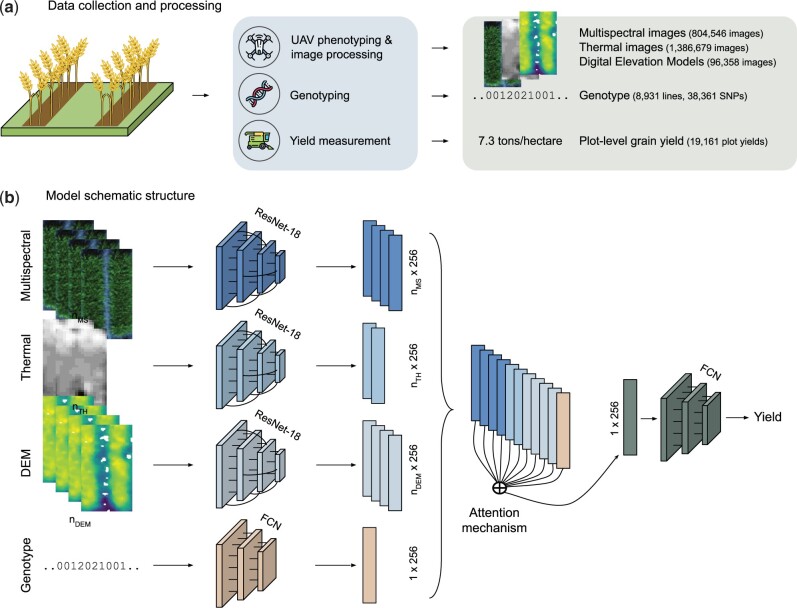
PheGeMIL leverages multi-channel inputs, combining dynamic phenotypic observations with genotypes. (a) Advanced spring wheat (*Triticum aestivum* L.) breeding lines from the International Maize and Wheat Improvement Center (CIMMYT) breeding program were sown during season 2017–2018. Multispectral and thermal images were collected by UAV flights over the experiment field and digital elevation model (DEM) developed from the images. Lines were profiled using genotyping-by-sequencing with calling of single nucleotide variants (SNPs). Final crop yield was measured by combine harvest in tons per hectare (t/ha) on a per plot basis. (b) The model flexibly enables the fusion of information across channels via a single deep learning architecture that is fully differentiable. Encoder networks for each data type transform images or genotypes into vectors of fixed dimensionality (256 dimensions). The attention mechanism then combines these vectorial representations in a single 256-dimensional vector, independently of the number of input images provided. The unique representation is then fed in a final fully connected network to predict plot-level yield. *n*_MS_, number of multispectral images for a given sample; *n*_TH_, number of thermal images for a given sample; *n*_DEM_, number of digital elevation model images for a given sample; ResNet-18, a convolutional residual neural network architecture for image feature extraction; FCN, fully connected neural network architecture; SNPs, single nucleotide polymorphisms.

We base our approach on the work of Ilse *et al.* (2018) and extend it to fuse multispectral and thermal images, temporal, spatial, and genomic information. We therefore treat each observation of a given plot as an instance of the object we aim to predict yield for. We rely on a deep neural network to encode the genotypic information and on ResNet architectures to encode the images for each plot. We then combine the obtained representations in a permutation-invariant MIL setup, that allows for efficient aggregation of data from diverse sources across time. We therefore obtain embeddings for each instance (i.e. data source) we are dealing with and we combine them using an attention-based aggregation function, described in Equations (2) and (3). Moreover, since some of our data sources consist in multiple, irregularly-sampled observations through time (e.g. unbalanced dataset) and since permutation-invariant aggregation can be efficiently used to learn on time series with irregularly spaced observation (Horn *et al.* 2020), we also applied the attention pooling framework along the temporal dimension. More practically, for each sample (i.e. each wheat plot), we have a final yield value and four data sources that need to be combined: multispectral images, thermal images, digital elevation models (DEM), and SNP array data. Moreover, for the first three data sources, each plot has multiple observations through time: from early images at the beginning of the growth process to images just before harvest. We therefore rely on a deep learning architecture that (i) takes each instance of every data source and transforms it into a fixed-size embedding via a data source-specific encoder, (ii) combines the obtained embeddings into a unique vectorial representation of the sample, (iii) computes the predicted grain yield for that sample. Figure 1b summarizes the architecture in a schematic view. The model can then be trained in an end-to-end fashion and learn the weights for the encoding, the pooling, and the prediction networks. To do so, we use a mean squared error (MSE) loss between the measured yield *y* and the predicted yield $\hat{y}$: $L_{\mathrm{MSE}}=\sum_{i=1}^{N}{\left({\hat{y}}_{i}-y_{i}\right)}^{2}/N$. Moreover, since the number of instances for each plot and data source is not constant, we can easily handle samples with less or more images as well as with missing data sources. We implemented our method in a flexible manner to be able to add and remove data sources easily. We rely on the PyTorch library (Paszke *et al.* 2019) to implement the model architecture, on PyTorch Lightning wrapper to speed up experiments (Falcon and The PyTorch Lightning Team 2019), and make our code available on GitHub (https://github.com/BorgwardtLab/PheGeMIL). For image-based data channels, we rely on a small residual network architecture *(*ResNet-18; He *et al.* 2016) given the relative simplicity of the images (small size). For genotypic information (SNPs) we use a FCN with two layers of 1024 and 512 hidden units respectively. We then force all embedding representations to a 256-dimensional vector and combine them using the attention mechanism. We employ a multi-head attention mechanism with *n* heads, meaning that we have *n* different combined vectors that we then concatenate and pass through the final fully connected layer of the model for the final regression. We experiment with a temporal encoding to also use the datestamp of each image acquired by embedding the dates as one-hot vectors and appending those to the embeddings generated by the residual convolutional networks. In a similar fashion we test channels encoding where we append a one-hot encoding of the channel to the 256-dimensional embeddings, to see if nudging the attention mechanism by indicating which data sources it is dealing with is helping. Due to memory constraints on the computing infrastructure, we cannot use all images for each instance (some instances have up to 200 images). We therefore add a parameter denoted as bag size which indicates a maximum number of images to randomly sample for each channel at every iteration. This means that throughout training, the set of chosen images for samples with many input images constantly changes. We then tune this hyperparameter with other ones in our setup.

### 2.4 Experimental design

For all phenotype prediction tasks, we do a 5-fold cross-validation on the entry (e.g. genetically unique breeding line), stratifying by trial. This ensures that there are no replicates of the same entry that can be both in the training and in the test set, which would upwardly bias the prediction accuracy. We then split the training set using the same stratification and obtain a validation set for the deep learning models. We use 80% of the data for training, 10% for validation, and 10% for testing. To guarantee comparability, the baseline models that do not require validation data can use it as training data, the test set are therefore the same for all compared methods and splits. Hyperparameters for the other baseline models are tuned via internal cross-validation on the training set (90% of the dataset) using the dedicated scikit-learn python library (Pedregosa *et al.* 2011). The high-dimensionality of the images impedes their direct usage in the baseline models, we therefore extract moments (mean and mode) of individual channels as well as calculated vegetation indices for each image. The vegetation indices considered are the normalized difference vegetation index (NDVI), the normalized difference red edge index (NDRE), and the green normalized difference vegetation index (GNDVI), and they are obtained as follows: where Red, RedEdge, NIR, and Green are the channels captured by the multispectral camera. Moreover, the baselines models are not capable of handling multiple instance input for the image channels: they can only handle a fixed-size input and concatenation of values across dates and instances is not possible, as the number of images per plot constantly changes. To tackle this, we average the above-mentioned values across images of a given plot. We do this both on a date-basis, where we group dates in four temporal groups: (i) 18 January 2018–31 January 2018, (ii) 01 February 2018–02 March 2018, (iii) 03 March 2018–10 March 2018, and (iv) 11 March 2018–21 March 2018.


$$\begin{array}{c} \text{NDVI}=\frac{\text{NIR}-\text{Red}}{\text{NIR}+\text{Red}},\\ \text{NDRE}=\frac{\text{NIR}-\text{RedEdge}}{\text{NIR}+\text{RedEdge}},\\ \text{GNDVI}=\frac{\text{NIR}-\text{Green}}{\text{NIR}+\text{Green}}, \end{array}$$

We then train the baseline model using individual date group values or using all the values combined. Each sample therefore has 16 features (2×5 channels and 2×3 VIs) for a given date group and 64 in the case of training with all dates. For the PheGeMIL model, we fine-tune the hyperparameters via a random search of 20 runs on a split using the validation’s Pearson correlation coefficient to select the best set of parameters. The tuned hyperparameters are:

1. Learning rate: the initial learning rate, chosen among $\left\{10^{-5},10^{-4},10^{-3}\right\}$
2. Learning rate scheduling: this parameter allows us to have scheduled changes in the learning rate throughout training. This has proven to improve training, we try to have no scheduling, a plateau scheme which reduces the learning rate once learning stagnates or a cyclic cosine annealing scheme (Loshchilov and Hutter 2017) chosen among {none,plateau,cosine}
3. Batch size: the amount of samples processed in parallel, chosen among {8,16}
4. Bag size: the maximum number of processed images for a given channel, chosen among {8,16,32}
5. Number of attention heads: the number of attention mechanisms used in parallel, chosen among {1,4,8}
6. Temporal encoding: whether temporal encoding is performed, chosen among {True,False}
7. Channel encoding: whether channel encoding is performed, chosen among {True,False}

We evaluate the regression performance by evaluating mean absolute error (MAE), MSE, Pearson correlation coefficient, and the coefficient of determination R2 at the plot level. All experiments were run on a dedicated cluster running Ubuntu 14.04.5 LTS, with 16 CPUs **(**Intel(R) Xeon(R) CPU E5-2620 v4 @ 2.10 GHz**)** each with 8 cores and 24 threads, 128 GB of RAM, and 8 GPUs.

### 2.5 Prediction in new environment

To predict yield on a new unseen environment, we preprocess the data for the new environment so as to obtain inputs of the same size that the ones used during training. We solely focus on multispectral images and genotypes and adjust the SNP selection to account for differences in typed mutations. PheGeMIL is therefore trained on the five folds described above of the first environment and evaluated on the full dataset of the new environment. For genotype-only predictions, PheGeMIL is trained using the multispectral images and genotypic information and fine-tuned using genotypes alone.

## 3 Results

We started by collecting phenotypic and genotypic information from individual wheat plots. We focus on the main remote sensing data sources easily accessible to breeding programs, namely 5-channel multispectral and thermal imaging. We also include digital elevation models (DEM), a representation of the plant height derived from the UAV-acquired images. Images were acquired at different time points during growth, with irregular time intervals between flights. We additionally obtained SNP marker data from genotyping-by-sequencing of the breeding lines. Finally, individual plot yield was measured. Figure 1a schematically describes the data acquisition procedure. To verify the ability of our method to generalize to new experiments, we also included data from a separate trial, from an entirely different environment and with a different plot type and agronomic management (raised beds versus flat) (Table 1).

**Table 1.** Details of the datasets after quality control and filtering.

		2018 YT	2018 EYT
Study	Location	Ciudad Obregon	Ciudad Obregon
	Condition	Two raised beds	Sown in flat
	Design	*α*-Lattice design in two blocks with plot size of 1.7 × 3.4 m^2^	Trial-specific *α*-lattice design in three blocks with plot size of 1.3 × 4 m^2^
Plants	# plots	19 161	3510
	# trials	320	39
	# entries	9596	1170
	# genetically unique entries	8931	1094
Multispectral images	# images	804 546	291 114
	# unique dates	14	4
	Avg # images per plot per date	8.36	20.73
Thermal images	# images	1 386 679	NA
	# unique dates	4	
	Avg # images per plot per date	36.18	
DEM images	# images	96 358	NA
	# unique dates	14	
	Avg # images per plot per date	1.00	
Genotypes	# typed SNPs	38 361	40 767

PheGeMIL relies on the Multiple Instance Learning (MIL) framework, for which a target value for a sample is predicted from its instances, where the number of instances can vary from sample to sample. In our case, we consider an individual plot as a sample, and all its observations (temporal images, DEM, and genotypes) as instances of that sample. To accommodate our multi-modal inputs, we leverage deep representation learning and attention, a recent deep learning mechanism that allows for efficient combination of latent representations. A schematic view of our model can be found in Fig. 1b. The flexibility of this approach allows us to use PheGeMIL with any combination of inputs, both during training and testing time.

**Figure 2:**
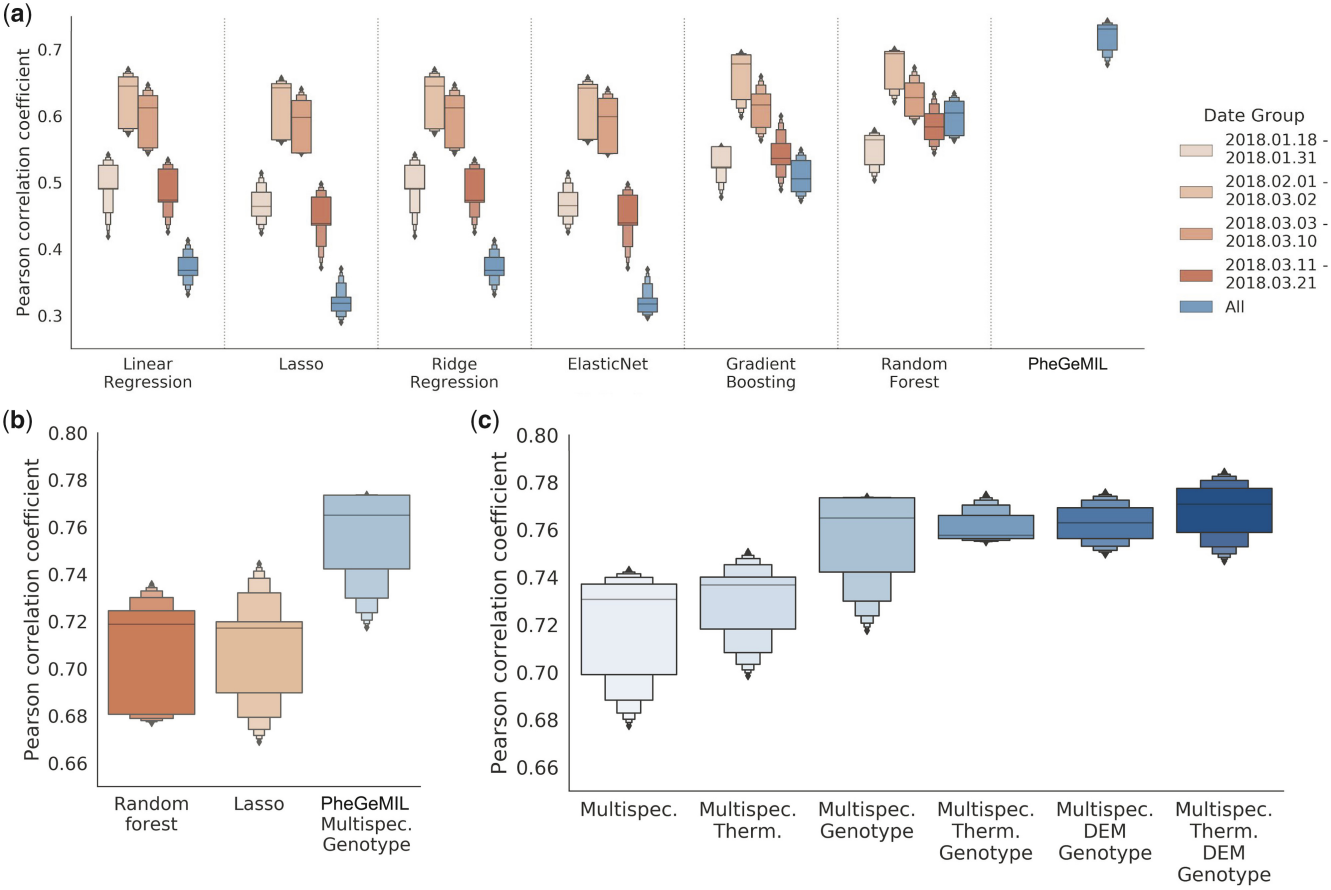
Performance of deep multiple instance learning (MIL) and genomic selection methods models for yield prediction measured using Pearson correlation coefficient over a 5-fold cross validation scheme. (a) Performance when relying on multispectral images alone. The baseline models use summary statistics of the multispectral channels and of notable vegetation indices computed on the images. Moreover, they are trained on images from different date groups, as images of late-growth stage carry more predictive power. (b) Performance when relying on a combination of multispectral and genotypic input for yield prediction for a nonlinear baseline (Random forest), a linear baseline (Lasso), and PheGeMIL. For the two baseline models, the most informative images were preselected and used for prediction (dates 01 February 2018–02 March 2018, as opposed to all for PheGeMIL). (c) Performance evaluation when adding new data channels in PheGeMIL prediction. Multispectral images are always kept in the comparison, given that they are the basis of the approach. Only a subset of the possible channel combinations is explored, as the focus lies in combining images and genotypic information. MIL, multiple instance learning; DEM, digital elevation models; Multispec., multispectral; Therm., thermal.

We initially assess the ability of our method to leverage raw multispectral image data without relying on popular transformations (e.g. VIs), on which most implementations of yield prediction from remote sensing are based. In particular, we compare the MIL with linear and nonlinear baselines trained using the same set of images (see Section 2.4). PheGeMIL clearly outperforms the baselines, both linear and nonlinear ones (Fig. 2) and has the benefit of not having to collect images at specific growth stages, as all images can be easily aggregated. The Pearson correlation coefficient between predicted and observed of 0.717 ± 0.028 for our MIL compared to 0.671 ± 0.037 for Random Forest at the second time point, the very best performing of the other model (optimally selected *post facto*). We can therefore state that PheGeMIL can be efficiently used to combine multiple observations of images across time, particularly for the large, dynamic, and unbalanced datasets common to field evaluation in breeding programs.

We then wish to evaluate the impact of adding other data sources. We start by incorporating genotypic information (SNP markers) in the training and evaluation procedure, both for our method and for a linear and a nonlinear competitive baseline: Lasso and Random Forest. Due to the massive dimensionality of the SNP array data (38 361 features) we only consider the go-to model Lasso regression, with its L1 regularization acting as a feature selector. For the comparison, we further gave an advantage to the baseline models by only preselecting images that are most informative (date group 2, 01 February 2018–02 March 2018), a situation that would not be possible in real-world implementations. For PheGeMIL, we used the attention-based aggregation to combine the embeddings from images across all dates together with the one obtained from the genotype fully connected network (FCN). Here again, our approach outperforms the linear and nonlinear baseline by a considerable margin (Fig. 2b) The observed Pearson correlation coefficient was 0.754 ± 0.024 for this new MIL compared to 0.707 ± 0.027 for Random forest and 0.708 ± 0.029 for Lasso. Furthermore, these results, when compared to the performance obtained by genotype-only based models (Pearson correlation coefficients of 0.559 ± 0.050 for Lasso, 0.335 ± 0.046 for a simple 3-layer fully connected network), also confirm that phenotype prediction can be greatly improved when incorporating covariates that model the environmental effects in the training population. We finally integrate the data from all four channels. Combining information from all channels is expected to improve the predictive performance of the algorithm: both thermal images and digital elevation models have been shown to be partially predictive of the final yield of the plots (Singh *et al.* 2019). This was confirmed as we observed that as more channels were incorporated into the MIL, the predictive abilities increased with a Pearson correlation coefficient of 0.767 ± 0.019 for the MIL model using all channels (Fig. 2c). The largest gain was provided by incorporating genotypic information, the gains then seem to saturate.

**Figure 3:**
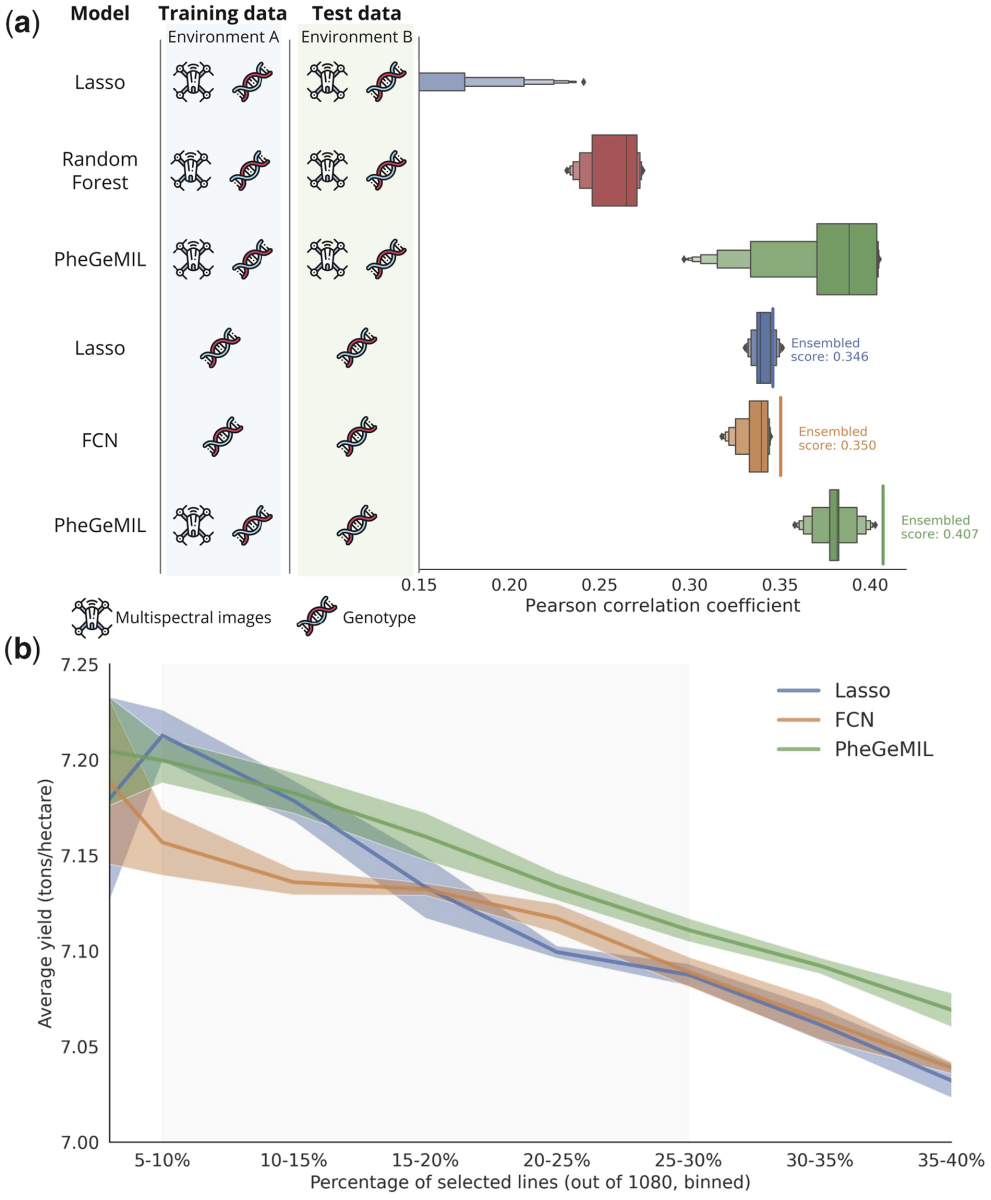
Generalization performance on a new environment and utilization for crop selection. (a) Comparison of yield prediction performance of a linear baseline (Lasso), two nonlinear baselines (Random forest and FCN) and our model (PheGeMIL) for prediction on a new, unseen environment using genotypic or phenotypic data. Multiple scenarios are evaluated. In all cases, training is done on data from environment A (2018 YT, see Table 1) and testing is done on data from environment B (2018 EYT). A set of experiments is conducted by training and evaluating on both multispectral images and genotypic data (first three rows). A second set of experiments is conducted by evaluating on genotypes alone (last three rows), to mimic prediction before sowing in breeding program scenarios. For baselines, training and testing must be done on the same data types and training can only be done on genotype alone. PheGeMIL, on the contrary, is trained with phenotypic data too, while still being evaluated on genotypes alone, thanks to the MIL framework. Distributions represent the performance in terms of Person correlation coefficient obtained on models trained on the 5 different splits of the training set. Ensembled performance for genotype-only predictions represents the prediction performance obtained when averaging the predicted values for a given sample across the 5 trained models. (b) Average yield obtained from a prediction-driven line selection of varying sizes (binned) using rankings derived from the values predicted by different ensembled methods. Lines are selected based on the predicted yield, their effective yield is then averaged across the set of selected lines and reported for an increasing selection size, ranging from 5% to 40% of the lines in the test set. MIL, multiple instance learning; FCN, fully connected network.

To evaluate the practical applicability of our method, we needed to verify its performance when applied to new experiments. We therefore measure the predictive ability of PheGeMIL and baseline models when trained in one environment and used to predict yield on a new and previously unseen environment with entirely different set of breeding lines (Fig. 3a). As expected, the prediction accuracy results are lower than for intraenvironment prediction. Yet, PheGeMIL still performed better, using the combined signal between multispectral images and genotypic data by a strong margin when compared to Lasso and Random forest (Pearson correlation coefficients of 0.373 ± 0.045 versus 0.026 ± 0.120 and 0.257 ± 0.018 respectively). To better mimic current breeding program strategies, we also predict using genotype alone which would be implemented as genomic selection prior to any field evaluations. The results presented here are calculated on the plot-level. We provide genotype-level evaluations and a comparison with MegaLMM in Supplementary Fig. S1. The classic genomic selection models such as Lasso must be trained on genotypic data alone, but the MIL training framework implemented in PheGeMIL is able to leverage phenotypic observations at training time, yet uses only genomic information for prediction. To have the most rigorous comparison, we also used a deep fully connected network (with the same architecture as the genotype embedders of PheGeMIL) trained on genotypes alone. Generally, models have a lower prediction variance when only relying on genotypes. This is due to the large difference in the UAV signal between the training and testing images. Unexpectedly, the Lasso baseline is far better off when using genotype values alone as compared to when relying on multispectral images and genotypes, as if the difference in multispectral signal is too large between the two environments to carry any signal at an aggregated level after normalization. Nonetheless, PheGeMIL still outperforms both the linear and the deep learning baselines (Pearson correlation coefficients of 0.381 ± 0.016 versus 0.341 ± 0.008 and 0.336 ± 0.011 respectively) showing the additional model accuracy gained by incorportaing phenomic data in the model training. Moreover, the performance gain does not come from using deep learning on the genotypic information only (as FCN does not improve over Lasso) but from leveraging the phenotype information together with the genotypes during training. Given that we have five trained models from the cross-validation scheme, we also ensemble the predictions as this is known to yield better performance (Sagi and Rokach 2018), finally obtaining a 17.6% improvement for PheGeMIL over the linear baseline.

We then translated this obtained performance of the models to the applications in breeding programs for improved selection from the breeder’s perspective. This translates in practical selection gains. In Fig. 3b, we rank the 1080 lines of the new environment according to their predicted yield and compute the average actual yield of the top-N lines. We can see that for the window of a typical selection (where 10%–30% of the lines are selected), PheGeMIL-based predictions almost always recommend a better selection than the baselines, resulting in an average relative improvement of 1.5% in the breeding program selections for a selection intensity between 10% and 30% of the total lines.

**Figure 4:**
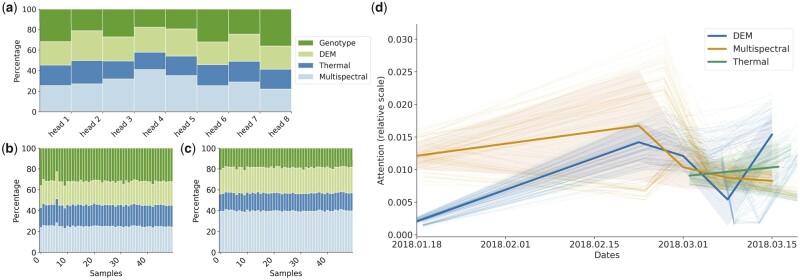
The predictions of the presented model can also be interpreted thanks to the attention mechanism. (a) Distribution of the attention given by the model to each of the input channels for a given sample: the distribution can be interpreted as the relative importance of each data source for the yield prediction of that sample. The selected architecture has 8 different attention heads, each represented by a column in the plot, and they all consider different aspects of the input, hence giving varying importance to each channel. On average, multispectral images is the channel most considered, indicating that the model prioritizes its input well, as multispectral images are known to better correlate with yield than the other modalities. (b and c) Attention of a heads 1 and 4 respectively for 50 samples, the attention is consistent across samples, indicating reproducible behavior. (d) The attention distribution can also be evaluated across time for images collected at different stages of the plant growth. The model focuses on multispectral images at the beginning of growth, multispectral, and Digital Elevation Models at mid-growth and thermal images at the end of the growth. Interestingly, the model gives more importance to images obtained at mid-growth, confirming results found in Fig. 1. MIL, multiple instance learning; DEM, digital elevation models; Multispec., multispectral; Therm., thermal.

Finally, we perform model interpretation on PheGeMIL. The attention mechanism contributes effectively to the performance of the model, but it can also be helpful to investigate the effects of the different input data. In fact, the attention values represent the relative contributions of each of the input instances to the final prediction. Therefore, we looked into these values to better understand (i) which data sources are more relevant, and (ii) which temporal windows contain the most informative (i.e. predictive) images. PheGeMIL relies on 8 attention heads, which behave differently and we need to investigate the attention value across all of them (Fig. 4a). When looking at the attention distribution across data channels (Fig. 4a), we observed that each head gives different relative importance to each channel in a consistent way across multiple samples (Fig. 4b and c). Multispectral images constantly received more attention (almost 40% of the attention in head 4), followed by genotypes. On the opposite side, thermal images are the ones receiving the least attention. This corroborates the findings of the ablation studies presented in Fig. 2c, where genotypes contribute more to the performance improvements. Since each input instance from the three image channels are also linked to a date, we can also look at the temporal distribution of the attention (Fig. 4d). When looking at these plots for all attention heads, one can observe a general trend for multispectral and DEM attention. The attention mechanisms deem earlier multispectral images as more important while it considers later DEM images as more relevant. This intuitively makes sense: at an early stage of growth, little information about the plant height and overall biomasss is contained in the DEM images. In comparison, relevant information about the soil properties, which could be visible at early stages when the crop canopy is not developed, can be accounted for in the multispectral images. Moreover, there was a higher cumulative attention load towards the middle the growth phase (e.g. 01 February 2018–02 March 2018), which coincide with the period for which linear and nonlinear baselines gave better results when using VIs from multispectral images (see Fig. 1a). These periods correspond to flowering and grain filling growth stages, critical timepoints for the ultimate determination of yield in wheat. Thermal images, on the other hand, do not seem to have considerably different importance across time. To ensure that thermal images are not confounding the relative importance at later stages of the other channels, we remove them at inference time but obtain similar results. Interpretability could be further studied at the genomic level by combining the attention study with guided backpropagation frameworks (Azodi *et al.* 2020, Talukder *et al.* 2021).

## 4 Discussion

We present PheGeMIL, a new deep learning architecture to efficiently fuse multi-channel and temporal data for yield prediction from multimodal datasets combining genomics and phenomics. We apply it to predicting wheat yield measurements across multiple experiments and show that it performs well in a variety of settings and outperforms currently used genomic seleciton models. Our approach offers several key benefits.

First, PheGeMIL enables the efficient combination of inputs across time, particularly without *a priori* knowledge of the most predictive features and timepoints. When looking at inputs from a single channel (i.e. multispectral images alone), the method clearly outperforms the baselines, both linear and nonlinear ones. This can be attributed to two phenomena: (i) the deep learning framework enables the use of the entire images instead of simple moments of their pixel value distributions; (ii) the multiple instance learning framework combined with the attention mechanism allows the efficient capture of inter-relationships between images of the same plot across observation points and time. The possibility to rely on images captured at any time during the plot growth offers considerable practical advantages: the data collection process needs not to be carefully planned for a limited window of time after seeding.

Second, PheGeMIL unlocks better performance by fusing data from multiple sources (multi-modal). We show that the predictive ability of the method increases with the number of input channels. In particular, we see a stark increase when fusing multispectral images and genotypic data (a 5% relative improvement as compared to multispectral-only, to a Pearson correlation coefficient of 0.754). These results, when compared to the performance obtained by genotype-only based models (Pearson correlation coefficients of 0.559 ± 0.050 for Lasso, 0.335 ± 0.046 for a simple FCN, a 35% relative improvement), also confirm that phenotype prediction can be greatly improved when incorporating covariates that model the environmental effects on the samples. A similar approach was suggested in the field of digital pathology, where histopathology images were combined with genomics features for prognostic prediction (Chen *et al.* 2022). The authors also report an improved performance of their model compared to unimodal approaches.

The performance of PheGeMIL keeps increasing with other input sources, although the gain seems to saturate: the addition of DEM and thermal images improves the performance only slightly (a Pearson correlation coefficient improvement from 0.754 to 0.767, i.e. 1.7%). DEM images are obtained from the multispectral images directly, it seems therefore plausible that part of their information content is already captured by the model when looking at the multispectral images. Nonetheless, the addition of image channels diminishes the variability of the performance, potentially indicating that the different sources corroborate each other and increase the model’s certainty. We can therefore conclude that the MIL approach combining four input channels is able to accurately predict the wheat grain yield of wheat crops. While comparison with other studies is difficult given the peculiarity of different crops and setups, we can observe that the performance we report is higher compared to the ones in other studies (Wang *et al.* 2014, You *et al.* 2017, Maimaitijiang *et al.* 2020).

Third, PheGeMIL acts as a powerful feature extractor for genotype-based prediction and improve performance even if the multi-modal input is only available on the training set. Our results suggest that attention-based deep learning approaches can efficiently extract nonlinear signals from genotypic inputs by leveraging ancillary phenotypes at training time. In fact, our method performs considerably better than the exact same architecture trained on genotypes (SNP markers) alone (14.9% relative improvement). This opens up exciting research directions, where combination of omics input and environment-dependent features could lead to more accurate predictive models. Furthermore, the attention mechanism itself could be extended to provide interpretability around genetic loci of interest and guide biomarker discovery.

Lastly, PheGeMIL, based on its improved predictive performance from genotypes alone, offers practical benefits for breeding programs to implement for genomic selection. We show that the predictions yielded by our method can lead to an average yield improvement of 1.5% over other prediction methods. While this improvement might seem marginal, it could be instrumental in closing the gap between the current average yield gains obtained by programs and the growth in demand for wheat across the globe by speeding up the breeding cycles with genomic and phenomic predictions.

## 5 Conclusion

We here introduced PheGeMIL, a flexible, accurate, and interpretable method to predict plant phenotypes from multi-model datasets and demonstrated its superior performance for wheat grain yield prediction. Our approach can be applied to widely diverse datasets and can incorporate numerous data channels with instances sampled at irregular time intervals. Additionally, we successfully combine genotypic information with environmental variables by fusing SNP array data with images that also capture environmental conditions such as soil properties and the effects of these conditions across time. We also show that PheGeMIL can efficiently leverage ancillary phenotypic observations to improve genotype-only predictions, opening up exciting directions for future work. This, in turn, allows PheGeMIL to better predict crop yield in an entirely new and unseen environment from genotype data alone, making it a potential tool to enhance efficient selection of crops during breeding programs.

## Supplementary Material

btad336_Supplementary_Data

## Data Availability

The associated image data and genotype data for this study are available in Dryad repository at doi*:* 10.5061/dryad.kprr4xh5p.
