# Supplementary material for: Multi-modal deep learning improves grain yield prediction in wheat breeding by fusing genomics and phenomics
Source: Bioinformatics. 2023 May 23;39(6):btad336. doi: 10.1093/bioinformatics/btad336 (PMC10246581; doi:10.1093/bioinformatics/btad336)
Supplement: btad336_Supplementary_Data [file btad336_supplementary_data.pdf]

Supplementary Material

1 Genotype-level predictions on the new environment

Figure 1 reproduces the results from the main text on the genotype-level. To account for trial effects, prediction targets were obtained by fitting a linear mixed model:

$$y_{ijk} = \mu + g_i + t_j + r_{k(j)} + e_{ijk} \tag{1}$$

where y_{ijk} is a trait value of genotype i within trial j and replicate k, μ is the mean, g_i is the fixed effect for genotype i, t_j is the random effect for trial j, $r_{k(j)}$ is the random effect for replicate k within trial j, and e_{ijk} is the residual effect. This procedure follows the approach in Krause *et al.* (2019), who reported a similar dataset. For models reported in the main text, predictions were then computed by averaging the plot-level predictions per genotype. MegaLMM was fitted on genotypic means obtained from a mixed model like above, directly resulting in genotype-level predictions. Parameters of MegaLMM were set as described for wheat yield prediction in Runcie *et al.* (2021).

References

Krause, M. R. *et al.* (2019). Hyperspectral reflectance-derived relationship matrices for genomic prediction of grain yield in wheat. *G3: Genes, Genomes, Genetics*, **9**(4), 1231–1247.

Runcie, D. E. *et al.* (2021). Megalmm: Mega-scale linear mixed models for genomic predictions with thousands of traits. *Genome biology*, **22**(1), 1–25.

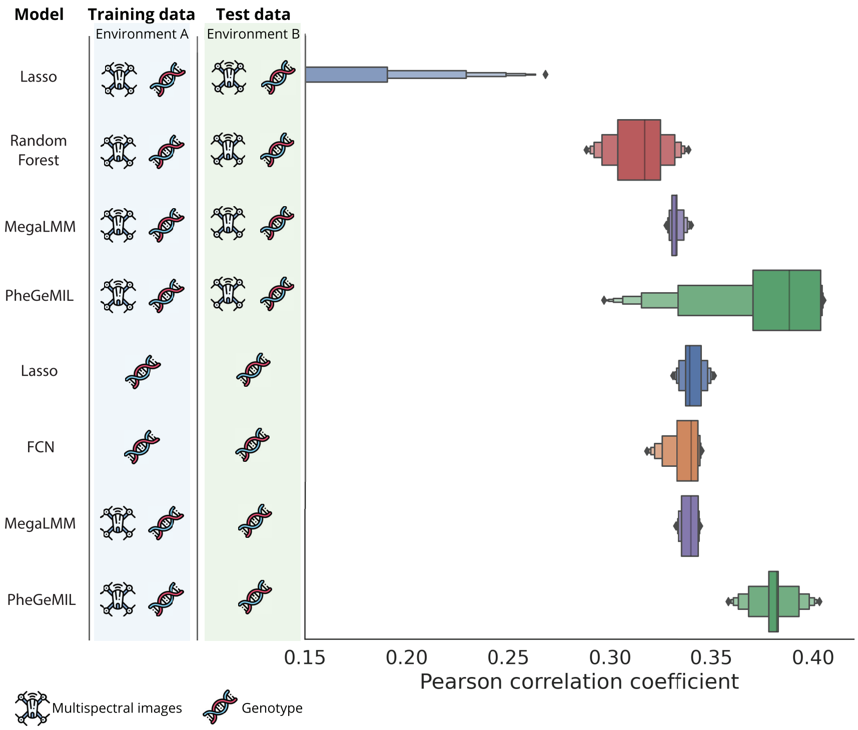

Figure 1: Generalization performance on a new environment and utilization for crop selection. **a** Comparison of yield prediction performance of a linear baseline (Lasso), two non-linear baselines (Random forest and FCN), MegaLMM, and our model (PheGeMIL) for prediction on a new, unseen environment using genotypic or phenotypic data. Multiple scenarios are evaluated. In all cases, training is done on data from environment A (2018 YT) and testing is done on data from environment B (2018 EYT). A set of experiments is conducted by training and evaluating on both multispectral images and genotypic data (first four rows). A second set of experiments is conducted by evaluating on genotypes alone (last four rows), to mimic prediction before sowing in breeding program scenarios. Distributions represent the performance in terms of Person correlation coefficient obtained on models trained on the 5 different splits of the training set.
